# Randomised Controlled Trial of Unsolicited Occupational Therapy in Community-Dwelling Elderly People: The LOTIS Trial

**DOI:** 10.1371/journal.pctr.0010002

**Published:** 2006-04-21

**Authors:** Anton J. M de Craen, Jacobijn Gussekloo, Gerard J Blauw, Charles G Willems, Rudi G. J Westendorp

**Affiliations:** 1 Department of Gerontology and Geriatrics, Leiden University Medical Centre, Leiden, Netherlands; 2 Institute for Rehabilitation Research, Hoensbroek, Netherlands

## Abstract

**Objective:**

The objective of this trial, the Leiden 85-Plus Occupational Therapy Intervention Study (LOTIS), was to assess whether unsolicited occupational therapy, as compared to no therapy, can decelerate the increase in disability in high-risk elderly people.

**Design:**

This was a randomised controlled trial with 2-y follow-up.

**Setting:**

The study took place in the municipality of Leiden in the Netherlands.

**Participants:**

The participants were 402 community-dwelling 85-y-old people, with a Mini-Mental State Examination score of >18 points at baseline.

**Interventions:**

Participants in the intervention group were visited by an occupational therapist who provided training and education about assistive devices that were already present and who gave recommendations and information about procedures, possibilities, and costs of assistive devices and community-based services. Control participants were not visited by an occupational therapist.

**Outcome Measures:**

The primary outcome measure was the score achieved on the Groningen Activity Restriction Scale. Secondary outcome measures included self-evaluations of well-being and feelings of loneliness.

**Results:**

The participants were evenly divided between the two groups: 202 participants were allocated to the intervention group and 200 participants to the control group. Of the 202 participants randomised to occupational therapy, 55 participants declined the proposed intervention. An occupational therapist indicated that of the remaining 147 participants, 66 (45%) needed an occupational therapy intervention. A total of 44 new assistive devices and five community-based services were implemented. During follow-up there was a progressive increase in disability in the intervention group (mean annual increase, 2.0 points; SE 0.2; *p* < 0.001) and control group (mean annual increase, 2.1 points; SE 0.2; *p* < 0.001). The increase in disability was not significantly different between study groups (0.08 points; 95% CI, −1.1–1.2; *p* = 0.75). There was also no difference between study groups for any of the secondary outcome measures.

**Conclusion:**

Unsolicited occupational therapy in high-risk elderly participants does not decelerate the increase in disability over time.

## Editorial Commentary

**Background:** Community-based services, such as occupational therapy, are widely used for elderly people to help them maintain independence. However, few studies have examined whether unsolicited offering of such services are effective in reducing disability, especially in the “oldest old”, that is, people 85 y or older.

**What this trial shows:** In this population of 85-y-olds, the effect of unsolicited occupational therapy was studied. The study consisted of therapists proactively making visits to elderly people in the trial, in order to provide additional services in addition to routine care. The researchers found no change in the reduction in disability over time in participants receiving unsolicited occupational therapy.

**Strengths and limitations:** The study is notable in that it provides good evidence, previously lacking, on the effects of unsolicited occupational therapy. The design was appropriate and randomisation was done correctly. Limitations include the fact that take-up of occupational therapy in the trial was not uniformly high.

**Contribution to the evidence:** Other evidence has shown that occupational therapy has beneficial effects in elderly people living at home. However, there has been little evidence about the effects of unsolicited occupational therapy in very elderly people, who are at particularly high risk of disability. This trial adds information from such a population and also indicates that making unsolicited visits, with the aim of finding people who have not already been provided with the services they need, is not likely to be of overall benefit.

## INTRODUCTION

Community-dwelling elderly people, and particularly the oldest old, are generally viewed as highly susceptible to the “inverse care law” [1]. That is, those in greatest need for preventive assessment and surveillance have the highest potential benefits, but are also the most likely to be missed. Hence, active case-finding and close follow-up might be an important strategy for maintaining the health, independence, and well-being of very elderly people, who are at a particularly high risk.

Assistive devices and community-based services are important tools in maintaining independence at old age. A recent systematic review on the efficacy of occupational therapy for community-dwelling elderly people found that occupational therapy in this age group has beneficial effects [2]. Apart from studies in younger elderly people, this carefully conducted review included only one small study in 85-y-olds who already had one or more disabilities in activities in daily living [3]. Hence, little is known about the possible benefits of occupational therapy in 85-y-olds.

We conducted a randomised controlled trial to assess whether unsolicited occupational therapy, compared to no therapy, can decelerate the increase in disability in a group of high-risk community-dwelling elderly people.

## METHODS

### Participants

We included 85-y-old participants using the infrastructure of the Leiden 85-Plus Study [4]. Participants who reached the age of 85 between March 2000 and May 2002, who were living in their own home, and who had a Mini-Mental State Examination (MMSE) [5] score above 18 points were eligible for inclusion in the study. First, a research nurse visited all possible participants in their own homes, explained that we were conducting a study on daily functioning and independence in old age, asked for informed consent, and performed all baseline measurements in consenting participants. Then, at the end of the visit, all participating individuals were randomly assigned in a 1:1 ratio either to the intervention group or to the control group. The control group received standard support, as routinely supplied by the social service system. Those assigned to the intervention group were asked for informed consent for the experimental part of the study. Participants in the control group were not informed about the experimental arm of the study. All participants in both the intervention group and the control group were revisited by the research nurse 6, 12, 18, and 24 mo after the baseline measurement. The whole study, including the informed consent procedure, was approved by the Medical Ethics Committee of the Leiden University Medical Centre.

### Interventions

Participants randomised to the intervention group were visited by an occupational therapist within 4 wk of randomisation. The methods by which the occupational therapists assessed the indication for intervention and implemented the assistive devices and community-based services have been described elsewhere [6]. In short, in their contact with the elderly people, the occupational therapists used the person–environment–occupation model as proposed by Law et al. [7,8]. This model uses a client-centred approach in which the occupational therapist clearly involves the client in the decision-making process. This approach was chosen since in this study participants did not consult the occupational therapist for an existing problem, but instead the elderly people were approached by the occupational therapist. The occupational therapist provided training and education about assistive devices that were already present. When indicated, participants received recommendations and information about procedures, possibilities, and costs of assistive devices and community-based services that might be beneficial for them. In cases in which participants decided to apply for the device or service, the occupational therapist provided assistance in filling out application forms or helped with purchasing. The occupational therapist focussed on assistive devices and community-based services in three main categories: mobility, meal preparation, and personal care. Text S1 includes a description of the most common items in each category.

### Measurements

Demographic details were collected for all participants. Income was categorised as low for participants who received a state pension only. Cognitive functioning was assessed with the MMSE [5].

The primary outcome measure was the score on the Groningen Activities Restriction Scale (GARS) [9,10]. The GARS is a unidimensional questionnaire that assesses restrictions in competence in activities of daily living. For example, questions are phrased as “Can you, fully independently, walk the stairs?” The score on each question has four categories, 1 being “yes, without difficulty”; 2, “yes, with some difficulty”; 3, “yes, with great difficulty”; and 4, “no, only with help from others”. In scoring the GARS, the use of assistive devices is allowed. We used the sum of the five GARS questions from the mobility category, the four items from the meal preparation category, and the four items from the personal care category as primary outcome measures. The minimum of the sum of the included GARS items is 13, indicating optimal performance in the three domains. Conversely, a GARS score of 52 indicates inability to perform any of the 13 items independently. Secondary outcome measures included the three subcategories of the GARS, the participant's well-being as assessed by Cantril's ladder [11], and loneliness as assessed by the de Jong-Gierveld questionnaire [12]. On Cantril's ladder, participants assess their own well-being by giving a mark between 1 and 10, where 1 is the worst possible well-being and 10 the best. The de Jong-Gierveld questionnaire consists of 11 items on perceived loneliness, where higher scores indicate more loneliness.

### Sample Size

We estimated that 86 participants in each group would be needed to detect a three-point difference (SD, 7 points) on the GARS (α, 5%; β, 80%). At age 85, a difference of three points in the GARS score represents a difference in functioning of about 2 y. The group size would need to be 193 to detect a two-point difference. We considered these calculations to be conservative since the primary analysis would be performed on repeated measurements.

### Randomisation Procedures

The randomisation sequence was generated with a computerised pseudorandom number generator and consisted of balanced blocks of size six. For each possible participant, the treatment allocation was inserted in a sealed envelope by administrative study personnel otherwise not involved in the trial. For participating individuals, the envelope was opened at the end of the baseline visit. For nonparticipating individuals, the envelope remained closed, something that was checked by the administrative study personnel after the return of the research nurse to the study centre. This procedure ensured concealed treatment allocation.

### Statistical Analysis

All cross-sectional association were assessed by cross-tabulation of dichotomous variables and by *t*-tests or the Mann–Whitney U test for continuous variables. Effects of the intervention at a single time point relative to control were assessed with a *t*-test on the difference between the outcome measure at that time point and the baseline measurement. The difference between intervention and control at all time points was assessed with linear mixed models. All analyses were performed using the intention-to-treat principle. *p*-Values of <0.05 were considered statistically significant.

## RESULTS

### Participant Flow

**Figure 1:**
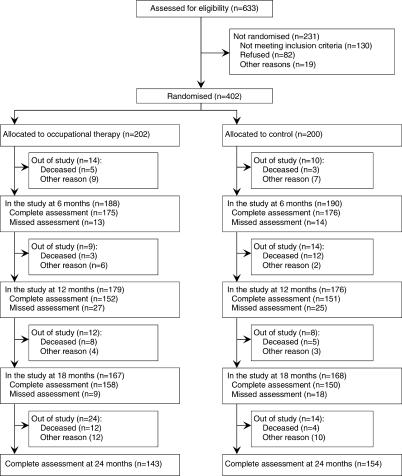
Flow of Participants through the Trial

Figure 1 shows the flow of the 633 participants who were assessed for trial eligibility. A total number of 231 individuals could not be randomised into the trial. The most frequent reason for nonparticipation pertained to the individuals' not meeting the inclusion criteria. Of the 402 participants who were randomised, 105 participants did not complete the trial, death being the main reason for not completing. Dropout was well balanced between the two study groups. Patients who did not complete the trial were similar to completers in terms of sex, income, and feelings of loneliness, but they were significantly more often living in a single household (74 of 105 versus 177 of 297; *p* = 0.048) and had worse functional performance (GARS scores of 20.1 versus 18.0; *p* = 0.003). These differences disappeared when we excluded the deceased participants from this analysis.

### Baseline Data

Table 1 shows the baseline characteristics for the 202 participants allocated to the intervention group and the 200 participants allocated to the control group. The groups were not statistically different on any of the baseline characteristics. In both groups, about two thirds were female, while about 60% were living in a single household.

**Table 1:**
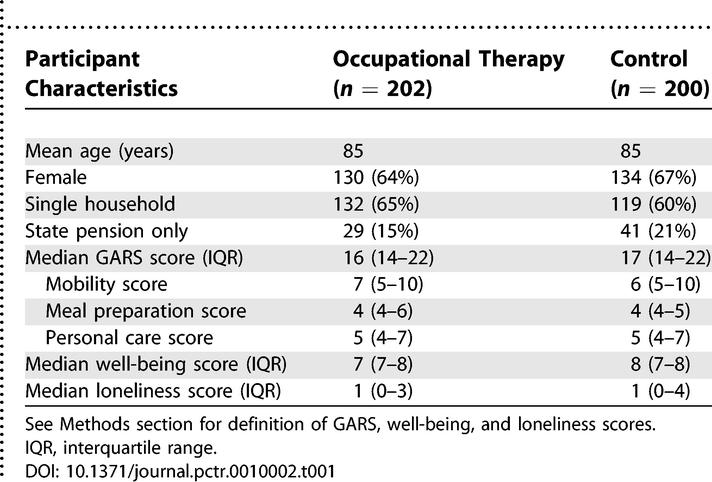
Baseline Characteristics of Study Participants

### Description of Occupational Therapy Intervention

Of the 202 participants randomised to occupational therapy, 55 participants declined the proposed intervention. This means they turned down the visit of the occupational therapist, although they agreed to stay in the study to perform the follow-up measurements. A description of all interventions that were provided to the 147 participants who were visited by the occupational therapist has been published elsewhere [13]. In short, 138 of the 147 participants (94%) already owned at least one assistive device in one or more domains. The total number of assistive devices was 591, of which 344 related to mobility, two related to meal preparation, and 245 related to personal care. The 147 participants used a total of 125 community-based services in the three domains (*n* = 52, 32, and 41, respectively). Moreover, as judged by the occupational therapist, 66 of the 147 participants (45%) needed an intervention in one or more domains: 50 participants needed one or more mobility-related interventions, six participants needed one or more interventions related to the preparation of meals, and 34 participants needed one or more interventions related to personal care. At the end of the study, the 66 participants who needed an intervention had obtained 44 new assistive devices and five community-based services.

### Study Outcome

Table 2 lists the increase in various outcome measures of study participants between baseline and 6 mo and between baseline and 24 mo. There was a decrease in performance after 6 mo, as indicated by an increase in GARS score, in both the intervention group (2.1 points; SE 0.4; *p* < 0.001) and the control group (1.8 points; SE 0.4; *p* < 0.001). The 0.3-point difference between the two groups was not statistically significant (95% CI, −0.7 to 1.40; *p* = 0.56). There was also no difference between study groups for any of the secondary outcome measures. Moreover, all differences between study groups at 24 mo were also not significant (Table 2).

**Table 2:**
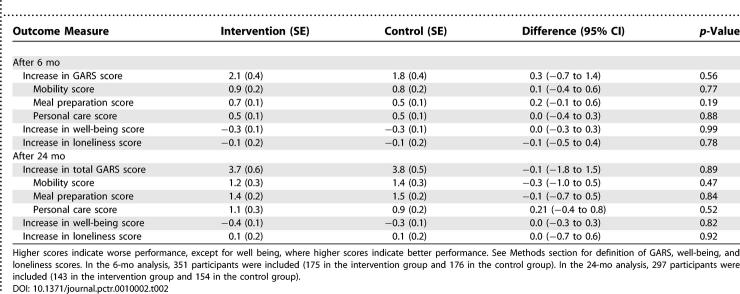
Outcomes in the Intervention Group and the Control Group 6 mo and 24 mo after Inclusion

**Figure 2:**
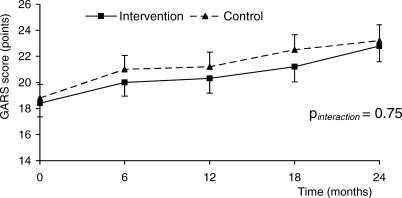
Increase in Disability in Intervention and Control Group during 2 y of Follow-Up Data points were estimated with linear mixed models. Data are presented as means with 95% confidence interval (CI). The increase in disability (GARS score) was significant for both intervention group (mean annual increase, 2.0 points; SE 0.2; *p* < 0.001) and control group (mean annual increase, 2.1 points; SE 0.2; *p* < 0.001). The increase in disability was not significantly different between study groups (0.08 points; 95% CI −1.1 to 1.2; *p* = 0.75).

Figure 2 shows the estimated increase in the GARS score over the whole study period using linear mixed models. Again, GARS scores increased progressively in both study groups, with no difference between intervention and control group. During follow-up, there was a progressive increase in disability in the intervention group (mean annual increase, 2.0 points; SE 0.2; *p* < 0.001) and control group (mean annual increase, 2.1 points; SE 0.2; *p* < 0.001). The increase in disability was not significantly different between study groups (0.08 points; 95% CI, −1.1 to 1.2; *p* = 0.75).

## DISCUSSION

We performed a large randomised controlled trial to assess whether unsolicited occupational therapy in high-risk community-dwelling elderly people can decelerate the increase in disability. Although a substantial number of assistive devices and community-based services were implemented, the intervention did not reduce the decrease in functional performance.

### Interpretation

There may be a number of reasons why we found no beneficial effect of our intervention. First, of the 202 participants randomised to occupational therapy only, 147 participants agreed to receive the intervention. This means that 55 participants declined the intervention. Because we analysed our results on an intention-to-treat basis, these 55 participants might actually have diluted a possible beneficial treatment effect. However, excluding them from the analysis would have introduced bias because it is unlikely that the group of participants who declined the intervention is a random sample of the total group. Second, of the 147 participants who were visited by an occupational therapist, less than half actually had an indication for an intervention. This means that this group of 85-y-olds can be considered well equipped, although there is room for further improvement. Third, not all participants for whom an intervention was indicated complied with the proposed intervention. In most cases, those who did not comply either felt they were confronted with a problem for which they did not experience having a problem or felt it was not necessary to solve the problem. In either of these cases, no action was taken by the participants.

We have previously reported the results of an unsolicited auditory rehabilitation programme for 85-y-old community-dwelling elderly people with untreated severe hearing loss [14]. In that study, the majority of participants declined auditory rehabilitation because they did not perceive the use of a hearing aid as necessary in order to function on a daily basis. Those who expected benefits from a hearing aid had already obtained one. A similar reasoning probably holds for the present study, in which occupational therapy was offered to the same age group. Those elderly people who expected to benefit from a certain assistive device or community-based services probably had already obtained them. This became apparent in the distribution of assistive device and community-based services that were already present (591 and 125, respectively) versus the assistive device and community-based services that were obtained (44 and five, respectively).

During the study, 52 participants died. Based on the 2001 mortality figures of the Dutch Central Bureau of Statistics (http://www.cbs.nl), we computed that 82 deaths were expected in this age group during the 2 y of follow-up. Therefore, the total number of deaths during our study should be interpreted as low. This lower mortality was probably caused by our inclusion criteria of participants with good cognitive function who were living independently. Since death was equally distributed over the study groups, this is unlikely to have influenced our result. Moreover, we accounted for study dropouts, including deaths, by analysing our data with a linear mixed model.

### Generalisability

We think that the population included in our study is a good representation of the population that would have benefited from the intervention, had it been efficacious. This is clear from the inclusion criteria for the trial: participants reaching the age of 85, living in their own home, and with an MMSE score above 18 points. Moreover, of the 633 participants who were approached, 402 (64%) participated. For the 231 participants who did not participate in the trial, the reason given by the majority (*n* = 130) pertained to not meeting the inclusion criteria.

### Overall Evidence

A recent systematic review of the efficacy of occupational therapy for community-dwelling elderly people found that occupational therapy is beneficial [2]. However, this systematic review included only one small study in 85-y-olds who already had one or more disabilities in activities in daily living [3]. To our knowledge, we here report the first randomised trial assessing the possible benefit of unsolicited occupational therapy in 85-y-olds.

## CONCLUSION

Traditionally, very elderly people who do not have the optimal number of assistive devices and community-based services to maintain their functional ability are considered exemplifications of the “inverse care law”. However, conditional on a well-organised healthcare system, very elderly people apparently do not solely depend on active case-finding and close surveillance. Instead, they seem to carefully plan the timing of their adaptations to maintain their functional abilities. We think that future elderly people will probably exhibit more of the phenomenon of making independent decisions on the use of assistive devices and community-based services. Therefore, we conclude that unsolicited offering of assistive devices and community-based services to high-risk 85-y-old participants does not decelerate the increase in disability over time.

## SUPPORTING INFORMATION

CONSORT Checklist (40 KB DOC)

Trial Protocol (66 KB DOC)

Trial Protocol Amendment (20 KB DOC)

Text S1 Assistive Devices and Community-Based Services (20 KB DOC)

## Abbreviations

GARS: Groningen Activities Restriction Scale
IQR: interquartile range
LOTIS: the Leiden 85-Plus Occupational Therapy Intervention Study
MMSE: Mini-Mental State Examination
