# Supplementary material for: Randomised Controlled Trial of Unsolicited Occupational Therapy in Community-Dwelling Elderly People: The LOTIS Trial
Source: PLoS Clin Trials. 2006 Apr 21;1(1):e2. doi: 10.1371/journal.pctr.0010002 (PMC1488896; doi:10.1371/journal.pctr.0010002)
Supplement: Trial Protocol — (66 KB DOC) [file pctr.0010002.sd002.doc]

**Promoting independent living**

**and well-being of the oldest old**

**A randomised controlled trial**

Protocol developed by

Dr. A.J.M. de Craen

Drs. J. Gussekloo

Dr. R.G.J. Westendorp

Promoting independent living and well-being of the oldest old

A randomised controlled trial

Responsible investigator R.G.J. Westendorp

Co-investigators J. Gussekloo, A.J.M. de Craen

Study location Department of General Internal Medicine

Section Gerontology and Geriatrics

Leiden University Medical Centre

PO Box 9600

2300 RC Leiden

The Netherlands

Tel: 071-5266640

Fax: 071-5248159

Contents

1 Summary 5

2 Introduction 6

3 Objectives 8

3.1 General objective 8

3.2 Specific objective 8

4 General features 9

4.1 Type of study 9

4.2 Duration of study 9

5 Participants 10

5.1 General 10

5.2 Inclusion criteria 10

5.3 Exclusion criteria 10

6 Intervention 11

6.1 Individual support trajectory 11

6.2 Informed consent 11

6.3 Allocation of intervention 12

7. Investigations 13

7.1 Baseline measurements 13

7.2 Follow-up measurements 13

8 Outcome 14

8.1 Primary outcome 14

8.2 Secondary outcomes 14

9 Statistical considerations 15

9.1 Sample size 15

9.2 Analysis 15

10. Ethical aspects 16

10.1 Declaration of Helsinki 16

10.2 Informed consent 16

10.3 Institutional Review Board 16

11 Study records 17

11.1 Case Record Form 17

11.2 Confidentiality 17

12 References 18

13 Appendix 19

1 Summary

Frail elderly in the community are especially susceptible to the 'inverse care law'. That is, those in greatest need, and for whom preventive assessment and surveillance have the highest potential benefit are most likely to be missed or to default. Active case-finding and close follow-ups of elderly persons with particularly high risk are especially important health intervention priorities.

Earlier surveys from the 'Leiden 85-plus study' have shown that a quarter of elderly persons aged 85 years loose their independence in few years time despite standard support of the social service system. Promoting independent living of elderly persons when assistive technology is implemented on an individual basis have not been studied as yet. This is especially important as implementing assistive technology may also have 'costs'. Feelings of loneliness and depression may increase as the living world becomes smaller and the number of social activities diminishes.

Embedded in the ongoing 1997-survey, we will perform a randomized controlled trial to test the hypothesis that an individual support trajectory, implementing assistive devices in daily activities, promotes independence and well-being of the oldest old.

Some 300 participants aged 85 years will be enrolled over a 18-months period and randomly assigned to an individual support trajectory or standard support from the social service system in a 1:1 ratio. In the individual trajectory an occupational therapist implements assistive devices based on an analysis of the home situation. Follow-up visits are scheduled 6-monthly for a mean of 3 years. The primary endpoint of the study is 'independence in the basic activities of daily living'. Secondary endpoints are the 'volume of (in)formal help', 'indication for institutionalized care', 'social functioning', 'depressive feelings', 'well-being' and 'loneliness'.

If the intervention proposed herein is proven to promote independence while well-being is maintained, a strong indication is obtained in which direction preventive measures can be developed.

2 Introduction

Our 1987-survey amongst the elderly population of Leiden showed that 20% of the people aged 85 years who lived in their own home were dependent on others in the activities of daily living (1). The number of elderly persons who lived at their own home fell with 24% between age 85 and 90 years. These data exemplify the loss of independence of the oldest old. The determinants of this loss of independence are under scrutiny in the ongoing 1997-survey 'Successful Ageing'.

'Personal care', 'mobility', and 'eating and preparation of meals' are basic activities of daily living that are critical to maintain independence when elderly people live in their own home. When the ability to perform these basic activities is lost, an indication for a house for the elderly or a nursing home is inevitable. From our 1997-survey it has become apparent that 74% of the people who lived in their own homes were only able to perform these basic activities with difficulties (2). This further illustrates that elderly people aged 85 years are at high risk of loosing their independence.

The unawareness of these disabilities and a reluctance to interfere among both elderly people and health-care professionals may contribute to the loss of independence. Several studies have shown that disabled people need, but do not own certain very basic and relatively inexpensive assistive technology (2-4). In line with international literature, we have found that less than 30% of the elderly with difficulties performing basic activities of daily living use walking aids, less than 10% have proper telephone adjustments, and less than 40% have a social alarm system (2).

From a preventive point of view, it is feasible to apply in-home assistive technology for elderly people (5). Tailor-made intervention programs may prevent them to become dependent on others. In regard to the implementation of assistive technology, two general mechanisms apply. First, it is feasible to change the attitude of elderly people towards assistive devices, hence increasing the use of these devices (6,7). Second, assistive devices for daily activities are not likely to replace professional home care when this care is already provided (8).

Studies that have evaluated the effects of implementing assistive devices in elderly populations have not been performed as yet. Hence, evidence that assistive devices promote independent living is not yet available. This is especially important as implementing assistive devices in an elderly population may also have 'costs'. Feelings of loneliness and depression may increase as the living world becomes smaller and the number of social activities diminishes.

The objective of the current study is to test whether an individual support trajectory, implementing assistive devices in daily activities, promotes independence and well-being of the oldest old.

3 Objectives

- 1. General objective

The general objective of this study is to investigate whether an individual support trajectory, implementing assistive devices in daily activities, promotes independence and well-being of the oldest old.

- 1. Specific objective

The effect of the individual support trajectory relative to standard support will be assessed by comparing the degree of ‘independence in the basic activities of daily living’ as measured with the Groningen Activities Restriction Scale.

4 General features

4.1 Type of study

This is a population based, randomised controlled study carried out in Leiden, the Netherlands. The study will be embedded in the ongoing population based 'Leiden 85-plus study'.

4.2 Duration of study

Total duration of the study will be four years.

5. Participants

5.1 General

The present intervention study will be embedded in the ongoing population based 'Leiden 85-plus study'. As such it will make use of the existing infrastructure to identify and invite elderly persons from the population living at home to participate. All will be visited at their own home.

5.2 Inclusion criteria

The following subjects are eligible for the study:

- inhabitants of the city of Leiden living in their own home
- reaching the age of 85 during the recruitment period.
- informed consent for the observational part of the study

5.3 Exclusion criteria

The following subjects will be excluded:

- a score of 18 or less on the Mini Mental State Examination (MMSE).

6 Intervention

6.1 Individual support trajectory

An occupational therapist will be responsible for planning and implementing the individual support trajectories for the participants who are randomly assigned to the intervention group. These individual support trajectories include a detailed analysis of the basic activities of daily living, a proposal for assistive devices, and a training scheme. Implementation of this trajectory will need an average of 2-3 home visits for each individual.

The following assistive devices are available for the individual support trajectories:

*Mobility*

- Standing up from bed (adjustable height bed, extender, bed rail, (bed)rope ladder)

- Standing up from chair (chair cushion, high chair, standing-up chair, lifting chair)

- Walking (walking aids, adapted shoes, electric outdoor transport, social alarm system, reimbursement of taxi costs)

*Personal care*

- Dressing (elastic shoe laces, buttoner, stocking applicator, special clothing, helping hand, dressing stick, shoe horn)
- Washing face and body (bath brush, shower stool, grab rail, non-slip bathmat, bathboard, specially towel)

- Going to the toilet (toilet raiser, raised toilet, toilet frames)

*Eating and preparing meals*

- Eating / drinking (adapted cutlery or service)

- Preparing food (work chair, handle tap, tap turner, jar/bottle opener, (electric) can opener, draining lid, non-slip mat, helping hand, meals on wheels)

- Shopping (shopping service, shopping trolley)

6.2 Informed consent

All participants of the Leiden 85-plus study are asked for informed consent to participate in the follow-up visits which are scheduled every six months. All baseline and outcome measures of this study (see section 7, investigations) are already routinely done in our current Leiden 85-plus study.

6.3 Allocation of intervention

Subjects who gave informed consent for the follow-up visits are randomly assigned in a 1:1 ratio to an individual support trajectory or to the standard support as currently supplied by the social service system. Those who are assigned to the individual support trajectory (the experimental arm of the study) will be asked informed consent for this part of the study. Subjects of the control group will not be informed of the experimental part of the study.

7 Investigations

7.1 Baseline measurements

At baseline the following items will be recorded:

- basic activities of daily living (Groningen Activities Restriction Scale)

- volume of formal help (information from care givers)

- volume of informal help (interview of participant and/or relatives)
- social functioning (Time Spending Pattern questionnaire)

- cognitive functioning (Mini Mental State Examination)

- depressive feelings (Geriatric Depression Scale)
- well-being (Cantrill's ladder)
- loneliness (de Jong-Gierveld questionnaire).

7.2 Follow-up measurements

Follow-up visits are scheduled every six months for a total period of three years. During each follow-up visit the follwing items will be recorded:

- basic activities of daily living (Groningen Activities Restriction Scale)

- volume of formal help (information from care givers)

- volume of informal help (interview of participant and/or relatives)
- indication for institutionalised care (information from care givers)
- social functioning (Time Spending Pattern questionnaire)
- depressive feelings (Geriatric Depression Scale)
- well-being (Cantrill's ladder)
- loneliness (de Jong-Gierveld questionnaire).

All instruments are presently used in our ongoing Leiden 85-plus study.

8 Outcome

8.1 Primary outcome

The primary outcome is the degree of ‘independence in the basic activities of daily living’ as measured with the Groningen Activities Restriction Scale.

8.2 Secondary outcome

The secondary outcomes are 'volume of formal help' (information from care givers), 'volume of informal help' (interview of participant and/or relatives), 'indication for institutionalised care' (information from care givers), 'social functioning' (Time Spending Pattern questionnaire), 'depressive feelings' (Geriatric Depression Scale), 'well-being' (Cantrill's ladder) and 'loneliness' (de Jong-Gierveld questionnaire).

9 Statistical considerations

9.1 Sample size

The mean score ± SD on the the Groningen Activities Restriction Scale in the 1997 survey was 17.3 ± 7.0 points (n=233). To detect a three-point increase on this scale an estimated number of 86 subjects are needed (alpha 5%, power 80%). The group size is increased to 193 individuals to obtain a 90% power. This calculation is conservative because the primary analysis will be performed on repeated measurements which is more sensitive to show statistical significant results.

Each calendar year a total of 350 inhabitants of Leiden wil reach the age if 85 years. Some 290 of them live at home. Based on data from the ongoing population based Leiden 85-plus study a 80% response rate is anticipated. We therefore expect that 300 subjects can be included in one and a half year.

9.2 Analysis

The score on the Groningen Activities Restriction Scale in subjects with an individual support trajectory will be compared with the score of those who received standard support using repeated measures analysis of variance (after transformation of not-normal distributed data). The continuous variables '(in)formal help', 'social functioning', 'depressive feelings', 'well-being', and 'loneliness' will be analysed similarly. The 'indication for institutionalised care' will be analysed using a time to event analysis. Correction for an unequal distribution of potentially distorting determinants after randomisation, i.e. sex, income, education, and living status, will be performed by entering the determinants as co-variates in the analyses.

10 Ethical aspects

10.1 Declaration of Helsinki

This study will be conducted in full accordance with the principles of the "Declaration of Helsinki" (as amended in Tokyo, Venice and Hong Kong) and with the laws and regulations of the Netherlands.

10.2 Informed consent

The information in the informed consent is intended to give each participant a thorough understanding of the nature of the study and the cooperation required. In Appendix 1 the full Dutch version of the patient information is printed. The informed consent form will first be approved by the Medical Ethics Committee.

10.3 Institutional Review Board

This protocol will be submitted to the Medical Ethics Committee. Approval must be obtained before starting the study.

11 Study records

11.1 Case Record Form

For all participating subjects, a Case Record Form (CRF) will be completed. The CRF consists of a sequential set of instructions with provision for data recording..

11.2 Confidentiality

All patients are identified by a study ID-number. Names will not be passed to others than trial personnel. The investigator will ensure that patient anonymity is maintained. On CRFs or other study documents, patients will not be identified by their names but by an identification code. The investigator will keep a separate log of patient codes, names and addresses.

The Leiden 85-plus Study is registered with the Dutch ‘Registratiekamer’, object number 12859, ‘verplichtings’ number 255770-97. Data storage will comply with the ‘Privacy Reglement’ of the Leiden University Medical Centre.

12 References

1. Bootsma-van der Wiel A, Westendorp RGJ, Knook DL. Het dagelijks functioneren en zorggebruik onder mensen van 85 jaar en ouder. *Ned Tijdschr Geneesk* 1997; 141:2170-76.

2. Successful ageing in the oldest old. Leiden, Leiden 85-plus study. 1997.

3. George J, Binns VE, Gladen AD, Mulley GP. Aids and adaptations for the elderly at home underprovided, underused and undermaintained. *BMJ* 1988;296:136-66.

4. Edwards NI, Jones DA. Ownership and use of assistive devices amongst older people in the community. *Age Ageing* 1998;27:463-8.

5. Fabacher D, Josephson K, Pietruszka F, Linderborn K, Morley JE, Rubenstein LZ. An in-home preventive assessment program for independent older adults: a randomised controlled trial. *J Am Geriatr Soc* 1994; 42:630-8.

6. Sonn U, Davegardh H, Lindskog AC, Steen B. The use and effectiveness of assistive devices in an elderly urban population. *Aging* 1996;8:176-83.

7. Knibbe JJ, Hulshof NA, Stoop AP, Friel RD. "Kleine hulpmiddelen: Hulp voor bewoners en zorgverleners". Utrecht, NIVEL. 1998.

8. De Klerk MM, Huijsman R. Aids in general daily activities can hardly replace professional home care. Results of a study among 75-year-olds or older. *Tijdschr Gerontol Geriatr* 1997;28:27-33.

13 Appendix

# **Leiden 85-plus Studie**

### **Uitleg van de studie**

## Het doel van de studie

In deze studie bestuderen wij de zelfredzaamheid en het welzijn van mensen van 85 jaar en ouder. Belangrijke vragen zijn bijvoorbeeld: Welke mensen verliezen hun zelfredzaamheid tijdens het ouder worden? Wat zijn hier de oorzaken van? Is aan deze oorzaken iets te doen? Zijn hulpmiddelen van nut om zelfstandig thuis te blijven wonen? Is het welzijn van de ouderen afhankelijk van de zelfredzaamheid?

## De deelname aan het onderzoek

Alle inwoners van de gemeente Leiden die 85 jaar worden, nodigen wij uit deel te nemen aan deze studie. De deelnemers aan de studie worden thuis bezocht door een onderzoeker van het team. *U hoeft dus voor geen enkel deel van het onderzoek naar het ziekenhuis te komen.*

Uw deelname aan de studie is erg belangrijk. Wij doen daarom ons best om het onderzoek voor u zo plezierig mogelijk te maken. Wij houden daarom zoveel mogelijk rekening met uw wensen.

Wanneer u besluit niet deel te nemen aan de studie, dan heeft dit voor u geen enkele consequentie. Ook kunt u tijdens de studie uw medewerking intrekken.

## De inhoud van de huisbezoeken

De studie loop over een periode van 4 jaar. Ieder half jaar wordt u bezocht door een verpleegkundige van het onderzoek. Ieder huisbezoek duurt ongeveer 1 uur.

In het eerste huisbezoek neemt de verpleegkundige een interview af. De vragen in het interview gaan onder andere over uw woonsituatie, uw lichamelijke beperkingen, de zelfredzaamheid in het dagelijks leven, ontvangen hulp, gebruik van hulpmiddelen, tijdsbesteding, eenzaamheid en tevredenheid over het leven.

Ieder half jaar komt dezelfde onderzoeksverpleegkundige bij u op huisbezoek om na te gaan of uw zelfredzaamheid in het afgelopen half jaar is veranderd, en wat daar de redenen van zouden kunnen zijn.

## Bescherming van uw persoonlijke gegevens

Zodra de gegevens van het interview zijn verzameld, worden uw naam en adres gescheiden bewaard van de verzamelde gegevens. Uw gegevens blijven op deze manier anoniem. Wanneer wij belangrijke afwijkende resultaten vinden, zullen we deze met u bespreken.

## Wie voert het onderzoek uit?

De studie wordt uitgevoerd door artsen en verpleegkundigen van de Sectie Gerontologie en Geriatrie, afdeling Algemene Interne Geneeskunde van het Leids Universitair Medisch Centrum (LUMC, voorheen het Academisch Ziekenhuis Leiden))

**Samenwerking met anderen.**

De Leidse zorginstellingen (bijvoorbeeld Thuiszorg Groot Rijnland en verzorgingshuizen) werken mee aan deze studie. Ook bij hen zal de verpleegkundige informatie inwinnen. Dit gebeurt uiteraard alleen als u hiervoor toestemming heeft gegeven. Alle huisartsen in Leiden zijn op de hoogte van deze studie.

## Voor vragen

Voor vragen van u of van uw familie is de secretaresse van het onderzoek, mevrouw Ingrid van de Boon, op werkdagen bereikbaar via telefoonnummer 071-526 6640.

**Bezoek / postadres:**

Sectie Gerontologie en Geriatrie

Leids Universitair Medisch Centrum

Albinusdreef 2

Postbus 9600

2300 RC Leiden

**Effect van hulpmiddelen op zelfredzaamheid**

# **Uitleg van de studie**

#### Het doel van de studie

U gaat deelnemen aan de Leiden 85-plus Studie naar het behouden van zelfredzaamheid van mensen van 85 jaar en ouder. Voor het behouden van de zelfredzaamheid kan het gebruik van hulpmiddelen een grote rol spelen. Binnen de studie willen we daarom het effect bestuderen van het gebruik van hulpmiddelen op uw zelfredzaamheid in de komende jaren.

#### Huisbezoeken door de ergotherapeut

Omdat het belangrijk is om hulpmiddelen te gebruiken die op uw persoonlijke thuissituatie aansluiten, zal de ergotherapeut(e) van het onderzoek u bezoeken. Dit huisbezoek duurt ongeveer 1 uur. De ergotherapeut is speciaal geschoold om bij thuiswonende ouderen advies te geven over hulpmiddelen die uw zelfredzaamheid kunnen bevorderen. Hij / zij bespreekt vervolgens welke hulpmiddelen voor u behulpzaam kunnen zijn.

De ergotherapeut(e) draagt zorg voor het verkrijgen van de hulpmiddelen die u geadviseerd worden. Als dat nodig is zal hij/zij hierover overleggen met bijvoorbeeld uw huisarts of de gemeente. Op het moment dat uw nieuwe hulpmiddel(en) thuis aanwezig zijn, leert de ergotherapeut u deze te gebruiken. Hiervoor zijn één tot drie korte huisbezoeken nodig.

#### Huisbezoeken door de verpleegkundige

Het effect van het gebruik van de hulpmiddelen op uw zelfredzaamheid wordt gemeten in de halfjaarlijkse bezoeken door een verpleegkundige van de Leiden 85-plus Studie.

#### Deelname aan het onderzoek

Uw deelname aan de studie blijft erg belangrijk. Wij doen daarom ons best om het onderzoek voor u zo plezierig mogelijk te maken. Wij houden daarom zoveel mogelijk rekening met uw wensen. *Ook voor dit deel van het onderzoek hoeft u niet naar het ziekenhuis te komen.*

## Voor vragen

Voor vragen van u of van uw familie is de secretaresse van het onderzoek, mevrouw Ingrid van de Boon, op werkdagen bereikbaar via telefoonnummer 071-526 6640.

**Bezoek / postadres:**

Sectie Gerontologie en Geriatrie

Leids Universitair Medisch Centrum

Albinusdreef 2

Postbus 9600

# 2300 RC Leiden
