# Supplementary material for: Randomised Controlled Trial of Unsolicited Occupational Therapy in Community-Dwelling Elderly People: The LOTIS Trial
Source: PLoS Clin Trials. 2006 Apr 21;1(1):e2. doi: 10.1371/journal.pctr.0010002 (PMC1488896; doi:10.1371/journal.pctr.0010002)
Supplement: Trial Protocol Amendment — (20 KB DOC) [file pctr.0010002.sd003.doc]

Protocol Amendment 1

NWO registration number 014-91-006

LUMC/CME protocol number P92/99-5

Title: Promoting independent living and well-being of the oldest old

The objective of the study ”Promoting independent living and well-being of the oldest old” is to test whether an individual support trajectory, implementing assistive devices in daily activities, promotes independence and well-being of the oldest old. In the original grant application and protocol we stated that in 18 months a total number of 300 subjects would be included. Moreover, all subjects would be followed up until the end date of the study, October 2003.

Inclusion of study subjects started in February 2000. The anticipated number of 300 included study subjects was reached in November 2001. After careful deliberation with all parties involved we have decided to implement a change of protocol. We have decided to increase the total number of subjects from 300 to 400, while at the same time shortening of follow-up to 24 months for all subjects. This change will increase power of the study. The total number of home visits by the research nurse will be unchanged. This is illustrated by the following calculation.

Original situation: all 300 subjects minimum follow-up 24 months (5 visits each), the last 100 subjects 1 additional visit, the middle 100 subjects 2 additional visits, the first 100 subjects 3 additional vistits. Total number of visits: 1500 + 100 + 200 + 300 = 2100.

New situation: 400 subjects 5 visits each. Total number of visits: 400 x 5 = 2000.

19 February 2002

Dr. A.J.M. de Craen, project leader

Section Gerontology and Geriatrics, LUMC
