# Supplementary material for: Randomised Controlled Trial of Unsolicited Occupational Therapy in Community-Dwelling Elderly People: The LOTIS Trial
Source: PLoS Clin Trials. 2006 Apr 21;1(1):e2. doi: 10.1371/journal.pctr.0010002 (PMC1488896; doi:10.1371/journal.pctr.0010002)
Supplement: Text S1 — (20 KB DOC) [file pctr.0010002.sd004.doc]

## Appendix 1. Assistive devices and community-based services.

*Assistive devices:*

Mobility: orthopedic shoes, walking frame on wheels, walking frame, walking cane, adapted bicycle, wheelchair, electric wheelchair, scootmobile, crutches, grab rails, ramps, shopping trolley, bathtub lift, electric dooropener, raising chair (electric), raised bed, electrically maneuvred bed, raised furniture in general, stairs elevator, helping hand

#### Meal preparation: adapted cutlery, bottle openers

Personal care: stocking aid, long shoe horn, dressing aid, button aid, toilet chair, bath brushes, raised toilet, toiletraiser, urinal, seats in shower, adapted watertaps, adapted entrance to bath or shower, anti-slip bath mats, safety railings around the bed, bathtub-board

Other: adapted telephones, magnifying glasses, special cushions, social alarm systems, adapted pencils

*Community-based services*

Mobility: subsidised transport, special parking tickets

Meal preparation: Meals-on wheels

Personal care: home care, community-nurse, help in household

Other: day care, volunteer-help
